# Circulating microRNA-122, microRNA-126-3p and microRNA-146a are associated with inflammation in patients with pre-diabetes and type 2 diabetes mellitus: A case control study

**DOI:** 10.1371/journal.pone.0251697

**Published:** 2021-06-02

**Authors:** Fahime Zeinali, Seyed Mohsen Aghaei Zarch, Alireza Jahan-Mihan, Seyed Mehdi Kalantar, Mohammad Yahya Vahidi Mehrjardi, Hossein Fallahzadeh, Mahdieh Hosseinzadeh, Masoud Rahmanian, Hassan Mozaffari-Khosravi

**Affiliations:** 1 Department of Nutrition, School of Public Health, Shahid Sadoughi University of Medical Sciences, Yazd, Iran; 2 Nutrition and Food Security Research Center, Shahid Sadoughi University of Medical Sciences, Yazd, Iran; 3 Department of Genetics, Faculty of Medicine, Shahid Sadoughi University of Medical Sciences, Yazd, Iran; 4 Department of Nutrition and Dietetics, University of North Florida, Jacksonville, FL, United States of America; 5 Yazd Clinical and Research Center of Infertility, Shahid Sadoughi University of Medical Sciences, Yazd, Iran; 6 Yazd Diabetic Research Center, Shahid Sadoughi University of Medical Sciences, Yazd, Iran; 7 Department of Biostatistics and Epidemiology, Research Center of Prevention and Epidemiology of Non-Communicable Disease, School of Health, Shahid Sadoughi University of Medical Sciences, Yazd, Iran; Lewis Katz School of Medicine, Temple University, UNITED STATES

## Abstract

The prevalence of type 2 diabetes mellitus (T2DM) is increasing dramatically worldwide. Dysregulation of microRNA (miRNA) as key regulators of gene expression, has been reported in numerous diseases including diabetes. The aim of this study was to investigate the expression levels of miRNA-122, miRNA-126-3p and miRNA-146a in diabetic and pre-diabetic patients and in healthy individuals, and to determine whether the changes in the level of these miRNAs are reliable biomarkers in diagnosis, prognosis, and pathogenesis of T2DM. Additionally, we examined the relationship between miRNA levels and plasma concentrations of inflammatory factors including tumor necrosis factor alpha (TNF-α) and interleukin 6 (Il-6) as well as insulin resistance. In this case-control study, participants (n = 90) were allocated to three groups (n = 30/group): T2DM, pre-diabetes and healthy individuals as control (males and females, age: 25–65, body mass index: 25–35). Expression of miRNA was determined by real-time polymerase chain reaction (RT-PCR). Furthermore, plasma concentrations of TNF-α, IL-6 and fasting insulin were measured by enzyme-linked immunosorbent assay. Homeostatic model assessment for insulin resistance (HOMA-IR) was calculated as an indicator of insulin resistance. MiRNA-122 levels were higher while miRNA-126-3p and miRNA-146a levels were lower in T2DM and pre-diabetic patients compared to control (p<0.05). Furthermore, a positive correlation was found between miRNA-122 expression and TNF-α (r = 0.82), IL-6 (r = 0.83) and insulin resistance (r = 0.8). Conversely, negative correlations were observed between miRNA-126-3p and miRNA-146a levels and TNF-α (r = -0.7 and r = -0.82 respectively), IL-6 (r = -0.65 and r = -0.78 respectively) as well as insulin resistance (r = -0.67 and r = -0.78 respectively) (all p<0.05). Findings of this study suggest the miRNAs can potentially contribute to the pathogenesis of T2DM. Further studies are required to examine the reproducibility of these findings.

## Introduction

Diabetes mellitus, as a chronic metabolic disease, is characterized by hyperglycemia due to inadequate insulin production by pancreatic beta cells (T1DM) or insulin resistance (inability to respond properly to insulin)(T2DM) [1, 2]. Type 2 diabetes mellitus (T2DM) is known as a risk factor for a variety of micro- and macro-vascular complications including cardiomyopathy, nephropathy and amputation that increases the rate of mortality in T2DM patients [3]. Fasting blood glucose (FBG), hemoglobin A1c (HbA1c), oral glucose tolerance test (OGTT) and homeostatic model assessment for insulin resistance (HOMA-IR) are among the most common tests that have been used for screening and diagnosis of diabetes mellitus. However, these parameters can predict the development of T2DM only when disease manifestation in patients have already caused metabolic alterations. In this regard, biomarkers for early detection of T2DM and identification of individuals at risk of developing diabetic complications could potentially complement the use of these parameters [4, 5]. Identifying biological predictors involved in T2DM can disclose new biological pathways involved in this disease as well as early detection, and prognosis of this disease [6].

MicroRNAs (miRNAs) are conserved non-coding and intracellular molecules that inhibit translation of their target molecules by binding to the 3’-UTR region of the target molecules [7]. The miRNAs are synthesized by *RNA POL II* in the nucleus, where they are also processed by *RNAse III Dorsha* as well as *DGCR8* to the pre-miRNA molecules (70 to 100 nucleotides long) that are transferred to cytoplasm by exportin 5 (XPO5). In the cytoplasm, pre-miRNA is processed by *Dicer*, and finally a mature miRNA molecule with 20 to 25 nucleotides is produced [8]. Recently, it has been shown that these molecules are involved in pathological processes such as T2DM and cancer [6, 9, 10]. MiRNAs are stable molecules present in tissues and a number of body fluids such as blood where they are protected from endogenous ribonuclease (RNase) activity [11]. Access to miRNA is possible with non-invasive methods and can be tested by specific and sensitive methods such as quantitative polymerase chain reaction (qPCR) [12].

Chronic inflammation in insulin-responsive tissues is one of the most important causes of insulin resistance consistent with elevated reactive oxygen species (*ROS*) levels leading to the activation of stress-related signaling pathways which in turn activates protein kinases such as c-Jun N-terminal kinases (JNKs), protein kinase C (PKC), glycogen synthase kinase ***3 (***GSK-3), nuclear factor kappa B (NF-kB) and P38 mitogen-activated protein kinase (MAPK) signaling, which plays an important role in insulin resistance [13, 14]. There is an increasing evidence that miRNAs are major players in inflammatory processes and immune responses.

MiRNA-122 is mainly expressed in liver and its function in lipid accumulation has been verified. In one study, enhanced miRNA-122 expression contributed to the inflammation resolution via TNF-α and IL-6 suppression in a human liver organoid model [15].

In human genome, miRNA -126 is found on chromosome 9 within intron 7 of the *EGFL7* gene [16]. It serves as an angio-miRNA and is mainly expressed in endothelial cells [17]. MiRNA-126 by targeting *Spred1* (Sprouty-related EVH1 domain containing 1) can play a crucial role in inflammation resolution. Elevated expression of *Spred1* via suppressing miRNA-126 expression in diabetic patients would promote IL-6, TNF-α and ROS production and results in endothelial cell dysfunction [18].

MiRNA-146a is located on long arm of chromosome 5 [19]. *Bhatt et al*. found that miRNA-146a has a protective role against inflammation by targeting the genes involved in the inflammation process in rats. In addition, the absence of protective mechanisms in miRNA-146a-/- mice exacerbates diabetic nephropathy. These results suggest miRNA-146a as one of the latest anti-inflammatory miRNAs [20]. In addition, *Chen et al*. [21] found that inflammatory cytokines secretion is significantly reduced from miRNA-146a transfected dendritic cells. On the other hand, the role of two human microorganisms known as herpes simplex virus-1 (HSV-1) as well as *Bacteroides fragilis* in the activation of NF-κB (p50/p65) and the up-regulation of miRNA-146a was suggested by *Lukiw et al*. (2008). These microbiota contribute to the inflammation-mediated amyloidogenic neuropathology of Alzheimer’s disease (AD) [22]. These conflicting studies underscore the fact that further studies are needed to determine the role of miRNA-146a in inflammatory signaling in AD and its potential underlying mechanisms.

Therefore, the primary goal of this study was to examine the expression levels of miRNA-122, miRNA-126-3p and miRNA-146a in T2DM and in pre-diabetic patients and in healthy individuals and determine whether they can be utilized as reliable biomarkers for early diagnosis and prevention of T2DM complications., pre-diabetic and healthy individuals to determine their implication as novel biomarkers in early diagnosis of T2DM. The second goal of this study was to investigate the relationship between miRNAs and inflammatory factors such as TNF-α, IL-6 and insulin resistance to understand the role of miRNAs in the development of T2DM.

## Materials and methods

### Sample size

The required sample size was calculated according to a previous study [23] and based on Ct changes of miRNA-146a as the key factor for sample size calculation. The mean change in Ct was considered as the effect size. A mean difference in serum miRNA-146a level of 0.5 between the two groups (patients with T2D and healthy individuals) was aimed to be detected for a specified α of 0.05 and a study power of 80%. Based on the proposed formula for case-control studies [24], we reached a sample size of 30 participants in each group.

### Subjects

Participants (n = 90) were patients with either T2DM, pre-diabetic patients, or healthy individuals (n = 30/group). The classification was based on guidelines provided by American Diabetes Association (ADA) as follows [25]:

Healthy subjects: fasting plasma glucose (FPG) < 100 mg/dL (< 5.6mmol/L) and HbA1c <5.7% (< 39 mmol/mol)

Prediabetic subjects: FPG 100–125 mg/dL (5.6–6.9 mmol/L) and/or HbA1c: 5.7–6.4% (39–47 mmol/mol)

Controlled diabetic subjects: FPG ≥126 mg/dL (7.0 mmol/L) and/or HbA1c ≥6.5% (48 mmol/mol)

The inclusion criteria: 1) age: 25 to 65 years; 2) having no micro-vascular and macro-vascular complications (e.g. retinopathy, nephropathy, amputation) 3) lack of infectious and coronary artery disease; 4) Body mass index (BMI) 25–35 kg/m2; 5) No smoking.

Healthy individuals were gender and age-matched with T2DM and prediabetes groups.

### Blood sampling

Fasting blood sample (10 ml) was taken from participants in the medical diagnostic laboratory of the Yazd Diabetes Research Center: 4 ml in tubes containing ethylene diamine tetra acetic acid (EDTA) for RNA extraction, 2 ml in a CBC tube containing EDTA to measure HbA1c and 4 ml in a test tube containing a clot activator for serum separation. dx.doi.org/10.17504/protocols.io.bax5ifq6 [PROTOCOL DOI].

### Biochemical measurements

Serum lipid profiles and fasting blood glucose (Pars Azmoon Kit) were measured by using an auto-analyzer (alpha-classical, Isfahan, Iran). The enzyme-linked immunosorbent assay (ELISA) was used to determine serum levels of fasting insulin (Monobind, Lake Forest, CA, USA. Cat# 5825-300A), TNF-α (Diaclone, Besancon, France. Cat# 950.090.096) and IL-6 (Diaclone, Besancon, France. Cat# 950.030.096). Homeostatic model assessment for insulin resistance (HOMA-IR) was calculated by the following equation: (fasting insulin (IU/ml) × fasting glucose (mmol/l)/22.5) [26].

### RNA extraction and quantitative real time PCR

Total RNA containing long RNA and small RNA were extracted by GeneAll Kit (General Biosystems, Seoul, Korea. Cat# 315–150) according to manufacturer’s instructions. The quality of RNA was assessed using Thermo Fisher Scientific nanodrop. The absorbance was measured at 260 and 280 nm wavelengths, and the RNA concentration was measured in each specimen. The A260/A280 provides a rough indication of purity of nucleic acid (RNA) [27]. Science the ratio of absorbance at 260 and 280 nm for samples were in the range of 1.8 to 2.2, we found out the purity of the isolated RNA. Then cDNA synthesis was performed by Stem-loop quantitative reverse transcription PCR (RT-qPCR). (Bonyakhteh, Tehran, Iran. Cat# BON209001). Finally, BONmiR QPCR Kit (Bonyakhteh, Tehran, Iran. Cat# BON209002) was used to investigate the miRNAs gene expression by Rotor Gene 6000 machine (Corbett, Concorde, NSW, Australia). The relative amounts of miRNAs (fold changes) were determined based on 2^-ΔΔCt^ (Livak) method. The changes in threshold cycle (ΔCt) were calculated by subtracting the Ct values of the reference SNORD from the Ct values of the target miRNAs (miRNA-122, miRNA-126-5p and miRNA-146a). ΔΔCt was then calculated by subtracting the average ΔCt values of the controls (healthy individuals) from the average ΔCt values of the cases (pre-diabetes or diabetes). ΔΔCt = (Ct _miRNA_−Ct _SNORD_) T2DM or pre-diabetes−(Ct _miRNA_−Ct _SNORD_) _healthy individuals_ [23, 28, 29].

To measure the expression levels of miRNA-122, miRNA-126-5p and miRNA-146a, qPCR in the whole blood sample of T2DM, pre-diabetic and control group was applied. SNORD was used as an internal control gene. Amplification efficiency has long been a predominant aspect of implementing real-time qPCR and plays a crucial role in the reliability as well accuracy. Therefore, amplification efficiency is calculated from the slope of the log-linear region, similar to that conducted for standard curves. Primer sets used in our study lie between 90–110% efficient [30].

### Bioinformatics analysis

Bioinformatics softwares such as *miRDB* (www.mirdb.org), *miRanda* (http://www.microrna.org/microrna/) and *TargetScan* (http://www.targetscan.org) were used to identify the potential targets (mRNAs) of miRNA-122, miRNA-126-3p and miRNA-146a.

### Ethical considerations

This study was approved by the Ethical Committee of Shahid Sadoughi University of Medical Sciences (IR.SSU.SPH.REC.1396.172). Written consent was obtained from patients who decided to join the project.

### Statistical analysis

All data are presented as mean ± standard deviation (SD). The data of this research were analyzed by Graph Pad Prism 7 software (Graph Pad, San Diego, CA, USA). The data normalization was performed using the Smirnov-Kolmogorov (K-S) test. The statistical significance of miRNA-122, miRNA-126-3p and miRNA-146a expression levels between T2DM, pre-diabetic and control group were investigated by one-way ANOVA. If significant, the *Tukey’s Post Hoc* test was used to compare means among groups. The association between inflammatory parameters as well as insulin resistance with miRNA-122, miRNA-126-3p and miRNA-146a expression levels were determined by Pearson’s correlation coefficient. P-values less than 0.05 were considered statistically significant.

## Results

The characteristics of participants are summarized in Table 1. Oligonucleotide sequences for RT-PCR analysis are shown in Table 2.

**Table 1:** Biochemical parameters of the studied groups.

	Healthy (n = 30)	Pre-diabetes(n = 30)	T2DM (n = 30)	P-value
	Mean±SD	Mean±SD	Mean±SD	
**FPG (mg/dl)**	94.37 ± 7.05	116.7 ± 6.51	138.2 ± 7.02	<0.001
**HbA1c (%)**	4.54 ± 0.2	5.9 ± 0.2	7.29 ± 1.22	<0.001
**Triglycerides(mg/dl)**	167.2 ± 87.6	178.6 ± 87.85	256 ± 385.5	0.44
**Cholesterol(mg/dl)**	162.2 ± 27.94	181.4 ± 44.36	199.1 ± 79.24	0.15
**HDL (mg/dl)**	39.82 ± 11.93	35.42 ± 12.07	34.07 ± 5.782	0.17
**Fasting Insulin (μIU/mL)**	10.47±0.81	11.26±0.98	12.06 ±1.29	<0.001
**HOMA-IR**	2.44± 0.27	3.25± 0.40	4.12±0.57	<0.001
**TNF-α(pg/ml)**	9.26±0.4	7.32±0.34	9.26±0.99	0.001
**IL-6(pg/ml)**	3.28±0.15	2.86±0.05	3.28±0.19	0.001
**Ct miRNA-122**	5.3±0.47	3.35±0.29	2.13±0.19	<0.001
**Ct miRNA-126-3p**	2.36±0.58	4.24±0.62	6.26±1.3	<0.001
**Ct miRNA-146a**	2.49±0.48	2.96±0.39	5.89±0.98	<0.001

**Table 2:** Oligonucleotide primers.

Gene	Sequence
*MiRNA*-122	Forward: GGAGTGTGACAATGGTGTT
*MiRNA* -126-3p	Forward: GCGTCGTACCGTGAGTAAT
*MiRNA* -146a	Forward: GAAGGTTGAGAACTGAAT
**SNORD**	Forward: GAACGATACAGAGAAGATTAG

### MiRNA-122 expression levels in T2DM, pre-diabetic and healthy control group

**Fig 1:**
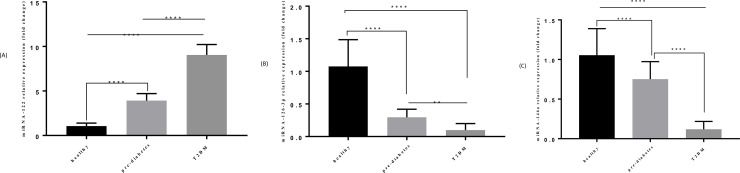
Comparison of expression levels of miRNA-122, miRNA-126-3p and miRNA-146a in T2DM (n = 30), pre-diabetic (n = 30) and control group (n = 30). A) Comparison of miRNA-122 expression level in 3 groups. B) Comparison of miRNA-126-3p expression level in 3 groups. C) Comparison of miRNA-146a expression level in 3 groups.

The expression levels of miRNA-122 in pre-diabetic (fold change = 3.92 ± 0.78) as well as T2DM individuals (fold change = 9.04 ± 1.17) were considerably up-regulated compared to healthy control group (fold change 1.05 ± 0.34) (Both p<0.001). Furthermore, the expression level of miRNA-122 was higher in T2DM individuals compared to pre-diabetic group (p<0.001) (Fig 1A).

### MiRNA-126-3p expression levels in T2DM, pre-diabetic and healthy control group

MiRNA-126-3p expression levels were decreased in patients with T2DM (fold change = 0.09 ± 0.09) compared to the control group (fold change = 1.07 ± 0.40) (p<0.001). Additionally, significant reduction was found in the pre-diabetic group (fold change = 0.29 ± 0.12) compared to the control group (P<0.001). Moreover, the expression level of miRNA-126-3p was lower in T2DM individuals compared to pre-diabetic group (P<0.001) (Fig 1B).

### MiRNA-146a expression levels in T2DM, pre-diabetic and healthy control group

The expression levels of miRNA-146a in T2DM individuals (fold change = 0.11 ± 0.09) as well as pre-diabetic (fold change = 0.75 ± 0.22) were considerably down-regulated compared to control (fold change 1.05 ± 0.33) (Both P<0.001). Furthermore, miRNA-146a expression level was lower in T2DM patients compared to pre-diabetic individuals (P<0.001) (Fig 1C).

### Identification key target gene

We identified that TNF-α and IL-6 are the potential targets of miRNA-126-3p and miRNA-146a. The results suggested that there is a miRNA-146a binding site in IL-6 and TNF-α genes. MiRNA-122 binding sites were not found in TNF-α, IL-6 genes. Other important targets are displayed in Table 3.

**Table 3:** Potential targets (mRNAs) for miRNA-122, miRNA-126-3p and miRNA-146a.

MiRNA	Potential target	Function
**miRNA-122**	Interleukin 1 receptor type 1	An important mediator involved in many cytokine-induced immune and inflammatory responses.
	REL proto-oncogene, NF-kB subunit	This gene belong to a family that Members of this family regulate genes involved in apoptosis, inflammation, the immune response, and oncogenic processes.
	PRKAB1	A regulatory subunit of the AMP-activated protein kinase (AMPK). AMPK is a heterotrimer consisting of an alpha catalytic subunit, and non-catalytic beta and gamma subunits. AMPK is an important energy-sensing enzyme that monitors cellular energy status.
**miRNA-126-3p**	Insulin receptor substrate 1	This gene encodes a protein which is phosphorylated by insulin receptor tyrosine kinase. Mutations in this gene are associated with type II diabetes and susceptibility to insulin resistance.
	SPRED1	Act as a homodimer or as a heterodimer with SPRED2 to regulate activation of the MAP kinase cascade.
	TRAF6	This protein functions as a signal transducer in the NF-kappaB pathway that activates IkappaB kinase (IKK) in response to pro-inflammatory cytokines.
	IL-6	Interleukin 6 is a potent pleiotropic cytokine that regulates cell growth and differentiation and plays an important role in the immune response.
**miRNA-146a**	TNF alpha induced protein 3	The protein encoded by this gene is a zinc finger protein and ubiquitin-editing enzyme, and has been shown to inhibit NF-kappa B activation as well as TNF-mediated apoptosis. The encoded protein, which has both ubiquitin ligase and deubiquitinase activities, is involved in the cytokine-mediated immune and inflammatory responses
	TNF receptor associated factor 3	This protein participates in the signal transduction of CD40, a TNFR family member important for the activation of the immune response. This protein is found to be a critical component of the lymphotoxin-beta receptor (LTbetaR) signaling complex, which induces NF-kappa B activation and cell death initiated by LTbeta ligation
	IL-6	Interleukin 6 is a potent pleiotropic cytokine that regulates cell growth and differentiation and plays an important role in the immune response.

### Association of inflammatory factors with circulating miRNA-122, miRNA-126-3p and miRNA-146a

A positive association between miRNA-122 and IL-6 (r = 0.83, P<0.001), TNF-α (r = 0.82, P<0.001) were observed. On the other hand, negative association were detected between miRNA-126-3p and miRNA-146a expression levels and IL-6 (r = -0.65 and r = -0.78 respectively) and TNF-α (r = -0.7 and r = -0.82 respectively) concentration (All P<0.001) (Table 4).

**Table 4:** The association between miRNA-122, miRNA-126-3p and miRNA-146a expression levels with inflammatory factors and insulin resistance.

	miRNA-122		miRNA-126-3p		miRNA-146a	
	R	P-value	R	P-value	R	P-value
**IL-6**	0.83	< 0.0001	-0.65	< 0.0001	-0.78	< 0.0001
**TNF-α**	0.82	< 0.0001	-0.70	< 0.0001	-0.82	< 0.0001
**HOMA-IR**	0.8	<0.0001	-0.67	< 0.0001	-0.78	<0.0001

### Association of insulin resistance with circulating miRNA-122, miRNA-126-3p and miRNA-146a

A positive correlation between miRNA-122 and insulin resistance (r = 0.8, p < 0.0001) was observed. Conversely negative correlation between miRNA-126-3p and miRNA-146a expression levels and insulin resistance (r = -0.67 and r = -0.78 respectively) were observed (P<0.001) (Table 4).

## Discussion

In this population-based, case-control study, we found a significant direct association between inflammatory cytokines including IL-6 and TNF-α as well as HOMA-IR with gene expression levels of pro-inflammatory miRNA-122 in general population. This significant association was reversed in terms of two other anti-inflammatory miRNAs. Such that both miRNA-126-3p and miRNA-146a gene expressions were inversely associated with IL-6 and TNF-α serum concentrations; and HOMA-IR in our study population. To the best of our knowledge, this study is the first observational study examining the association between inflammation-related miRNAs with serum levels of inflammatory cytokines, in particular IL-6 and TNF-α; and HOMA-IR in T2DM patients. Evaluation of these relations is more relevant to develop diagnostic and therapeutic methods of chronic diseases including T2DM [31]. Growing evidence has shown a link between specific miRNAs and chronic inflammation in T2DM. The inflammatory state has crucial role in hyperglycemia insulin resistance of these patients [32]. Increasing levels of inflammatory cytokines specially TNF-α are at the heart of inflammatory cascades linked to insulin resistance [33], as we observed in present study in which levels of TNF-α and IL-6 were significantly higher in diabetic and pre-diabetic patients compared to healthy controls. Our findings were in line with other reports that have shown the elevated levels of TNF-α in serum of T2DM patients [34–36]. Similarly, in terms of IL-6, increased serum concentrations of IL-6 were seen in diabetic patients by other previous studies [37, 38]. Based on experimental studies, up-regulation of TNF-α and IL-6 induces insulin resistance through activation of JNK and MAPK signaling pathways and consequently disrupts insulin signaling pathway leading to insulin resistance [39, 40]. As our other findings, a significant positive association between miRNA-122 and TNF-α and IL-6 was seen. Moreover, in the current study, elevated gene expression of circulating miRNA-122 was observed in T2DM patients. This finding confirms the results of earlier works indicating that the miRNA-122 expression levels can be used as a powerful biomarker in diagnosis of T2DM [41–44]. The role of T lymphocytes, a major component of the immune system, as pathogenic mediators in T2DM is well-described [45]. Valentina *Manfe et al*. identified miRNA-122 in advanced cutaneous T-cell lymphoma (CTCL). They also identified that enhanced expression of miRNA-122 triggers Akt phosphorylation and activation [46]. It can be shown that miRNA-122 may play a crucial role in elevated expression of inflammatory factors via Akt activation. The study also showed that miRNA-122 concentration is correlated with insulin resistance which is consistent with previous studies [44, 47]. MiRNA-122 may target PRKAB1, a subunit of AMPK, which is a key regulator of IR in muscle [47, 48]. Although the possible role of miRNA-122 in modulation of inflammatory cascades is well described, the exact mechanism through which miRNA-122 affects TNF-α and IL-6 levels consequently linked to insulin resistance in T2DM patients remains poorly understood. Future studies are needed in order to clarify the exact mechanisms of action between miRNA-122 and IL-6, and to see their precise correlation as well as the key role of miRNA-122 in the presence of inflammation in T2DM.

We found an inverse significant relation between gene expression levels of anti-inflammatory miRNA-126-3p and miRNA-146a and serum concentrations of TNF-α and IL-6; as well as HOMA-IR. In addition, the decreased gene expression levels of miRNA-126-3p and miRNA-146a were observed in both pre-diabetic and T2DM patients. These findings were in consistent with others which have reported the reduced levels of miRNA-126 in diabetic and pre-diabetic patients compared to healthy individuals, that can be used as a potential biomarker in early detection of T2DM even in patients with nephropathy and coronary artery disease [49–51]. Elevated expression of miRNA-126 as an anti-inflammatory miRNA, decreased the secretion of TNF-α as well as IL-6 in human gingival cells [52]. It has been suggested that miRNA-126 regulates inflammatory cytokines secretion through inhibiting TRAF6 and hampering NF-κB signaling pathways [53]. It seems that these observations along with the results of this study which showed a negative association between the expression of miRNA-126-3p and HOMA-IR, supports the anti-inflammatory role of miRNA-126-3p. Consistently, *Noha A*. *Rezk et al*. showed a negative, but not significant, association between serum miRNA-126 and insulin resistance [4]. Overall, it may suggest that miRNA-126 would improve the insulin resistance through regulating NF-κB signaling and secretion of inflammatory biomarkers including TNF-α and IL-6 [45]. Despite of the crucial contribution of NF-κB signaling in clarification of a link between miRNA-126 and the inflammatory cytokines release, it was not examined in the present study and future studies are needed to elucidate underlying mechanisms. In terms of miRNA-146a, our findings were in line with the findings of previous studies in which demonstrated that reduced levels of miRNA-146a were associated with insulin resistance, some pro-inflammatory cytokine genes and circulatory levels of inflammatory markers in T2DM patients [54]. However, the results of primary studies are controversial. In contrast to the findings of the present study, some studies failed to find any significant change in miRNA-146a levels [49], and another study has reported the significant increase in circulating miRNA-146a in pre-diabetics [55]. A most recent meta-analysis has concluded that gene expression of miRNA-146a in whole blood and PBMs elevates in T2DM subjects but not in serum and plasma [56]. It seems that some sex and age differences in circulating miRNAs might adjust these controversial findings [57]. Moreover, it might be due to different genetic variation in different population. We also found a negative correlation between miRNA-146a and IL-6, TNF-α, which is consistent with the findings in previous studies on other chronic and inflammatory conditions. Consistently, *Kamali et al*. reported an elevated expression of NF-kB (as a key activator of IL-6 and TNF-α) through miRNA-146a knockdown in human umbilical vein endothelial cells [58]. *Yang et al*. also reported that miRNA-146a suppression in human alveolar macrophages led to enhanced expression of IL-6 and TNF-α [59]. Cumulatively, it can be concluded that miRNA-146a negatively regulates the production of IL-6 and TNF-α. Moreover, our observation that miRNA-146a expression was negatively correlated with IR (insulin resistance) is aligned with previous studies. *Balasubramanyam et al*. [54] reported that reduced levels of miRNA-146a were associated with IR in Type 2 diabetic patients. Some studies have shown that miRNA-146a may reduce insulin resistance by activating JNK and P38 MAPK pathways which can play a major role in insulin signaling pathway [60, 61]. However, as we mentioned, more investigations are needed to extract the exact mechanism in this regard.

The present study has several strengths. This work was the first study that evaluated the association between three miRNAs and inflammatory factors as well as insulin resistance in T2DM, pre-diabetic and healthy individuals. Moreover, in order to minimize the variation in inflammatory status, only individuals with BMI 25–35 and non-smokers were included and all groups were matched for sex and age. However, some limitations are needed to be considered. Due to the case-control design of this study, causality cannot be inferred. In addition, we did not evaluate the effect of dietary antioxidants on inflammatory factors. Evaluating the insulin resistance by hyperinsulinemic euglycemic clamp as a gold-standard method instead of HOMA-IR index utilized in this study can help to confirm current findings. Moreover, assessing the effects of these three altered miRNAs on the expression of TNF and IL6 genes would be helpful to confirm our results. Conducting clinical trials with the larger sample size and using luciferase report assay, as a powerful tool for miRNAs target identification, can also be suggested for future studies.

## Conclusions

In summary, these results showed that the expression of miRNA-122 increased in T2DM patients compared to healthy individuals. Moreover, miRNA-126-3p and miRNA-146a expression levels decreased in T2DM individuals. Additionally, a significant relationship between miRNA-122, miRNA-126-3p, miRNA-146a and inflammatory factors and insulin resistance were observed in this research. MiRNAs may play a crucial role in pathophysiology of T2DM through inflammatory pathways. Their implications as preventative and diagnostic biomarkers need further investigation.

## Supporting information

S1 Table The demographic characteristics of the studied groups. (DOCX)

S2 Table Expression levels of miRNA-122, miRNA-126-3p and miRNA-146a in T2DM (n = 30), pre-diabetic (n = 30) and control group (n = 30). (DOCX)

## Abbreviations

ELISA: Enzyme-linked immunosorbent assay
PCR: Polymerase chain reaction
HOMA-IR: Homeostatic model assessment for insulin resistance
TNF-α: Tumor necrosis factor alpha
miRNA: MicroRNA
EDTA: Ethylene diamine tetra acetic acid
T2DM: Type 2 diabetes mellitus
HbA1c: Glycosylated hemoglobin type A1c
QPCR: Quantitative polymerase chain reaction
UTR: Untranslated region
IL6: Interleukin 6
IR: Insulin resistance
ROS: Reactive oxygen species
JNK: c-Jun N-terminal kinases
NF-kB: Nuclear factor kappa-light-chain-enhancer of activated B cells
MAPK: Mitogen-activated protein kinase
FPG: Fasting plasma glucose
