# Supplementary material for: Circulating microRNA-122, microRNA-126-3p and microRNA-146a are associated with inflammation in patients with pre-diabetes and type 2 diabetes mellitus: A case control study
Source: PLoS One. 2021 Jun 2;16(6):e0251697. doi: 10.1371/journal.pone.0251697 (PMC8171947; doi:10.1371/journal.pone.0251697)
Supplement: S1 Table — (DOCX) [file pone.0251697.s001.docx]

***The demographic characteristics of the studied groups***

|  | **Healthy (n = 30)** | **Pre-diabetes(n=30)** | **T2DM (n =30)** | **P-value** |
| --- | --- | --- | --- | --- |
| **Age (years) ^a^** | 55.37 ± 8.47 | 54.86 ± 6.38 | 53.03 ± 9.66 | 0.76 |
| **BMI ^a^** | 29.8 ± 2.89 | 30.17 ± 2.98 | 30.27 ± 3.11 | 0.82 |
| **Male ^b^** | 15 (50) | 15 (50) | 15 (50) | 1 |
| **Married ^b^** | 21 (70) | 23 (76.7) | 20 (66.7) | 0.68 |
| **University educated ^b^** | 15 (50) | 17 (56.7) | 14 (46.7) | 0.73 |
| **Presence of family history of diabetes ^b^** | 10 (33.3) | 12 (40) | 11 (36.7) | 0.87 |

^a:^ Mean±SD

^b:^ Frequency (percent)
