# Supplementary material for: Circulating microRNA-122, microRNA-126-3p and microRNA-146a are associated with inflammation in patients with pre-diabetes and type 2 diabetes mellitus: A case control study
Source: PLoS One. 2021 Jun 2;16(6):e0251697. doi: 10.1371/journal.pone.0251697 (PMC8171947; doi:10.1371/journal.pone.0251697)
Supplement: S2 Table — (DOCX) [file pone.0251697.s002.docx]

***Expression levels of miRNA-122, miRNA-126-3p and miRNA-146a in T2DM (n=30), pre-diabetic (n=30) and control group (n=30).***

| **Groups**  **Fold change** | **Healthy (n = 30)**  **Mean±SD** | **Pre-diabetes(n=30)**  **Mean±SD** | **T2DM (n =30)**  **Mean±SD** | **P(Value)** |
| --- | --- | --- | --- | --- |
| **MiRNA-122** | 1.05±0/34 | 3.92±0/78 | 9.04±1/17 | <0.001 |
| **MiRNA-126-3p** | 1.07±0/40 | 0.29± 0/12 | 0.09±0/09 | <0.001 |
| **MiRNA-146a** | 1.05±0/33 | 0.75±0/22 | 0.11±0/09 | <0.001 |
